# Agricultural governance to strengthen the multisectoral response to antimicrobial resistance: the experience of Bolivia

**DOI:** 10.3389/fvets.2026.1909859

**Published:** 2026-10-07

**Authors:** María de los Ángeles Gatica, Jorge Berrios, Pablo Willson, Carmen Bullon

**Affiliations:** 1 FAO Regional Office for Latin America and the Caribbean, Food and Agriculture Organization of the United Nations, Santiago, Chile; 2 FAO Bolivia, Food and Agriculture Organization of the United Nations, La Paz, Bolivia; 3 FAO, Food and Agriculture Organization of the United Nations, Rome, Italy

**Keywords:** agriculture sector, antimicrobial resistance, AMR governance, multisectoral coordination, One Health

## Abstract

Antimicrobial resistance (AMR) is considered by the World Health Organization (WHO) as one of the main threats to human health. The effective implementation of the One Health approach poses important governance challenges. Despite the international recognition of the need for multisectoral coordination to address AMR, progress in consolidating effective governance mechanisms remains limited. In Bolivia, despite attempts to establish a multisectoral committee and a National Action Plan on AMR (NAP), these have not been consolidated. In this context, the National Service for Agricultural Health and Food Safety (SENASAG) implemented the Committee for the Containment of Antimicrobial Resistance in Agricultural Health and Food Safety (CC-RAM), which made it possible to strengthen sectoral coordination, alignment of stakeholders, and the implementation of sectoral actions. A qualitative approach is used, based on the analysis of the institutional process of implementing a governance mechanism in the agricultural sector. The case study of Bolivia suggests that, in the context analyzed, agricultural governance may help create favorable conditions for strengthening efforts to address AMR within the sector and could contribute to the effective and sustainable functioning of multisectoral governance under a One Health approach.

## 1 Introduction

Antimicrobial resistance (AMR) is considered by the World Health Organization (WHO) as one of the main threats to human health (1). It is estimated that in 2019, AMR directly caused approximately 1.27 million deaths and was associated with around 4.95 million deaths worldwide (2). In addition, AMR generates significant economic and productive consequences (3). In the agricultural sector, antimicrobials play a key role in animal health, welfare, and productivity, contributing to food security, food safety, and public health (4).

In response to this problem, the international community adopted the Global Action Plan on AMR in 2015 (5), which recognizes that addressing AMR requires a One Health approach (6). This approach involves coordination among different sectors of human, animal, and plant health, as well as the environment, and the participation of multiple stakeholders (7).

However, the effective implementation of the One Health approach poses important governance challenges. Governance can be understood as the set of “formal and informal rules, organizations, and processes through which public and private stakeholders articulate their interests and make and implement decisions” (8). In this sense, governance goes beyond formal regulatory frameworks and involves the interaction among multiple institutions and stakeholders (8).

Strengthening governance may contribute to reducing the burden of AMR (9), and contexts of political or institutional instability may present greater difficulties for the implementation of effective governance mechanisms.

In the case of AMR, the challenge of multisectoral governance occurs at two levels.

On the one hand, the challenge occurs within the public sector, as authorities have a mandate limited to the competences granted to them by law, which prevents them from acting beyond those competences. In addition to this, the hierarchical nature of state bureaucracy does not facilitate coordination among public institutions, leading to coordination difficulties not only at the level of authorities with national competence (ministries, state agencies), but also between national and subnational authorities (e.g., regional and local levels).

On the other hand, the challenge occurs in the relationship between the public and private sectors, since the public sector is responsible for enforcing compliance with legislation by the private sector, which may be a source of distrust between both sectors and may hinder public–private coordination in joint participation spaces.

An additional challenge in addressing AMR is the need to adopt a One Health approach that takes into account not only the role of authorities responsible for public health, but also—under an equity perspective among sectors—the important role played by authorities responsible for animal health, plant health, and ecosystems (7).

Indeed, the strategy a country adopts to address AMR cannot be one that is exclusively focused on public health, since antimicrobials used in humans are also present in animal production systems, agricultural crops, and the environment. Therefore, any strategy to address AMR will be ineffective if it does not consider actions aimed at mitigating the generation and spread of AMR from and between animals, crops, and the environment.

Despite the international recognition of the need for multisectoral coordination to address AMR, progress in consolidating effective governance mechanisms remains limited. According to the global database for Tracking AMR Country Self Assessment Survey (TrACSS), by 2025, 91% of countries (from a total of 184 countries) had some form of multisectoral coordination system under a One Health approach (9% report not having one); however, only 22% of these are classified as sustained. In this context, although most countries have developed National Action Plans (NAPs), their actual implementation remains limited (10). In addition, the involved sectors have promoted measures to contain AMR; however, these actions are often developed in a fragmented manner, with limited coordination mechanisms among stakeholders, which makes it difficult to advance efficiently and sustainably in AMR containment (11).

Bolivia is not exempt from these challenges. Although the country initiated in 2016 the process of developing its NAP on AMR and the establishment of a multisectoral committee involving authorities responsible for human health, agriculture, and the environment, the absence of regulatory support guaranteeing its operability has limited the articulation of a coordinated and sustained national strategy to address this issue.

Faced with the need of the agricultural sector to identify alternatives to advance in the implementation of the AMR agenda, and given the limitations observed in multisectoral coordination, the National Service for Agricultural Health and Food Safety (SENASAG) identified as a viable strategy the strengthening of coordination within the agricultural sector, in order to organize its stakeholders, consolidate technical positions, and improve its internal coordination capacity. As a result of this process, the Committee for the Containment of Antimicrobial Resistance in Agricultural Health and Food Safety (CC-RAM or the Committee) was established through an Administrative Resolution of SENASAG.

This article presents the experience of the CC-RAM in Bolivia as a mechanism to strengthen the agricultural sector (understood in this article as encompassing animal health, plant health, and food safety, in line with SENASAG’s institutional mandate), which has contributed to improving its technical, representational, and operational capacity in addressing AMR, in a context of limited multisectoral coordination among the human health, agriculture, and environment sectors. Based on this experience, it proposes understanding agricultural sector governance as an element that may contribute to creating favorable conditions for the effective and sustainable functioning of multisectoral governance, without seeking to replace it.

## 2 Methods

### 2.1 Methodological approach

This article is situated within the project “Implementation of Codex standards to support containment and reduction of foodborne antimicrobial resistance” (ACT project), implemented by FAO and funded by the Republic of Korea.

The study adopts a qualitative approach based on a single case study design to analyze the experience of the CC-RAM in Bolivia, covering the period from its formalization in November 2023 through December 2025, which constitutes the data collection cut-off date for this analysis. The Committee has continued to operate beyond this date.

Four types of data sources were used. First, technical reports resulting from the sequential application of FAO AMR-related assessment tools between 2022 and 2023: the FAO Progressive Management Pathway for Antimicrobial Resistance (FAO-PMP-AMR), implemented in October 2022^1^; the FAO Assessment Tool for Laboratories and AMR Surveillance Systems (FAO-ATLASS), implemented in March 2023^2^; and the ACT tool, implemented in June 2023^3^. The results of these assessments were validated by the national counterpart of the project, SENASAG, prior to their use as inputs for this article. Second, institutional and regulatory documents, including SENASAG Administrative Resolution No. 383/2023, the General Animal Health Regulation (REGENSA), and the minutes of the 11 CC-RAM meetings held between April 2024 and December 2025. Third, the direct institutional engagement of the author team, who participated as part of the FAO technical assistance team supporting the design and implementation process of the CC-RAM alongside SENASAG. Fourth, published institutional materials documenting stakeholder perspectives on the CC-RAM, including a success story featuring interviews with participating stakeholders (12), and records from the presentation of the CC-RAM experience in FAO international fora (13).

Information from these sources was analyzed through a descriptive thematic synthesis, organized around a set of predefined analytical categories: process of creation, organizational structure and functioning, differentiating features, results achieved, success factors, and lessons learned. Documentary evidence was triangulated with the authors’ direct institutional engagement in the process, in order to provide a grounded and contextually informed account of the CC-RAM experience. The study also draws on an established governance dimensions framework (14), applied in the Discussion to interpret the CC-RAM’s experience.

This study did not involve new interviews or the collection of primary data from human participants. The stakeholder perspectives referred to above draw exclusively on previously published, publicly available materials. As no human participants were recruited for this study, no ethics committee approval was required.

Given this design, the evidence presented in this article should be understood as reflecting the institutional process of establishing and operating a sectoral coordination mechanism, rather than an independent evaluation of its outcomes on AMR-related indicators (further discussed in Section 5, Limitations).

### 2.2 Positionality statement

All authors participated while serving as FAO personnel or under FAO-supported technical arrangements. They were all directly involved, in different capacities, in the technical assistance process that supported the design and establishment of the CC-RAM within the framework of one specific FAO project (“Implementation of Codex standards to support containment and reduction of foodborne antimicrobial resistance”). This dual role—as facilitators of the process analyzed and as authors of this article—shapes the perspective from which this article is written.

This position offers the advantage of direct, contextually grounded knowledge of the process, which is not always accessible to external observers. At the same time, it entails a risk of bias toward a favorable interpretation of the CC-RAM’s implementation, given the institutional interest in the success of an initiative that FAO itself supported. Readers should therefore note that while factual elements of the account (e.g., dates, regulatory milestones, meeting frequency, composition of working groups) are anchored in documentary sources, the interpretation of success factors and lessons learned draws substantially on the authors’ direct institutional experience, complemented by stakeholder comments documented in institutional success stories and international dissemination events. This interpretation is presented as such, rather than as an independent assessment, and claims regarding the effectiveness of the CC-RAM in advancing AMR containment outcomes remain outside the scope of the evidence available (see Section 5, Limitations).

## 3 Assessment of policy/guidelines options and implications

### 3.1 AMR governance context in Bolivia

Bolivia has faced significant challenges in consolidating a governance mechanism to address AMR containment, particularly regarding multisectoral coordination.

Within the framework of the ACT project, an initial assessment phase was conducted to identify priorities and actions to be supported during its implementation phase (2021–2026).

As part of this diagnostic phase, between 2022 and 2023 the FAO-PMP-AMR, FAO-ATLASS and ACT tools were applied in Bolivia. These tools made it possible to identify, from the food and agriculture sector, gaps in legislation, governance, internal coordination, and monitoring of commitments undertaken for AMR containment, among other aspects.

At the time of the assessments, Bolivia did not have a general governance framework to regulate the cooperation among ministries on issues related to the One Health approach. Although the country had initiated the development of a NAP on AMR since 2016 and had established a multisectoral coordination mechanism, both faced limitations due to the lack of regulatory backing to ensure sustained operability and effective implementation capacity.

In this context, AMR governance was articulated on a temporary basis through a multisectoral coordination committee. A draft regulation had been developed to formally regulate the functioning of this committee, which envisaged, among other aspects, the possibility of establishing sectoral subcommittees (health, agriculture, and environment), allowing for more in-depth analysis of specific issues and the development of sectoral action plans aligned with the NAP. However, as with the NAP, it had not yet received tri-ministerial approval from the Ministry of Health and Sports, the Ministry of Rural Development and Land (now the Ministry of Productive Development, Rural Affairs and Water), and the Ministry of Environment and Water (now the Ministry of Development Planning and Environment).

As a result, this body lacked a formal allocation of human, financial, and technical resources for its operation. This situation was compounded by weaknesses related to difficulties in maintaining regular participation in meetings, the absence of clearly defined institutional responsibilities, and the lack of monitoring mechanisms to drive priority actions, including the formulation, approval, and formalization of the NAP.

Despite the institutional commitment expressed through the transitional committee and the technical advances achieved within the agriculture and food safety sector—such as the implementation of awareness-raising campaigns targeting different stakeholders, the existence of the Network of Official Food Analysis Laboratories (RELOAA), which includes AMR analysis in indicator pathogens in food for human consumption and the publication of corresponding reports (15), and the implementation of good production practices programs, among others—the absence of both a legally backed multisectoral committee and the approval of the NAP on AMR could have limited the country’s capacity to coordinate actions, build consensus on priorities, and strengthen national governance in this area.

In parallel, in 2020 the agricultural sector had incorporated the One Health approach into its own regulatory framework through its explicit adoption in the REGENSA (16).

In response to the need for the agricultural sector to advance in AMR containment, strengthening the sector’s internal organization was identified as a strategic priority through the creation of an agricultural coordination mechanism aimed at addressing the challenges related to this issue in a more structured manner within the sector itself.

### 3.2 Establishment of the CC-RAM

The establishment of the CC-RAM was the result of a prior technical diagnostic process in which FAO assessment tools (FAO-PMP-AMR, FAO-ATLASS, and the ACT Tool) were applied, identifying gaps in governance, regulation, and in the implementation of the NAP on AMR (17). Although the country had a draft NAP and a multisectoral committee under development, both lacked the regulatory backing necessary to ensure their consolidation and operationalization.

Based on these findings, it was identified that—without abandoning efforts to formally establish the Multisectoral Coordination Committee—there was a need and opportunity to create a coordination mechanism specific to the agricultural sector. This mechanism was expected to strengthen institutional articulation and engagement with the private sector, thereby contributing to a more effective approach in addressing AMR. This approach was consistent with the country’s tradition of public–private articulation as a driver of development in the agricultural sector.

In this context, in October 2023 SENASAG presented a draft regulation for the establishment of an agricultural sectoral committee. Subsequently, on November 21, 2023, the regulation on the functioning of the CC-RAM was officially approved through Administrative Resolution No. 383/2023 (18), thereby granting it legal backing.

The Committee was established as a sectoral coordination space that brings together public and private institutions linked to animal and plant health and food safety. Its structure includes a steering committee and technical working groups by production sector, responsible for promoting sectoral actions aligned with national priorities on AMR.

### 3.3 Objectives of the CC-RAM

Without abandoning efforts to strengthen multisectoral coordination in the country, the CC-RAM, as established by Resolution No. 383/2023, was conceived as a sectoral mechanism aimed at strengthening AMR governance in the areas of animal and plant health and food safety. Its main objective is to establish a formal space for discussion, coordination, and action, enabling the articulation and oversight of AMR-related actions in the food and agriculture sector, while promoting a systematic and comprehensive approach, without prejudice to SENASAG’s authority to adopt decisions within its mandate (18).

Likewise, the committee seeks to consolidate a consensus-based sectoral position that, once the multisectoral AMR committee is formalized, will enable organized and representative participation of the agricultural sector in the national multisectoral coordination body. In strategic terms, the CC-RAM is intended to contribute to strengthening the implementation of the NAP from the agricultural sector, promoting multisectoral coordination grounded in sectors that are previously organized and aligned.

### 3.4 Organization and functioning of the CC-RAM

The CC-RAM operates as a sectoral coordination mechanism led by SENASAG, the competent authority on matters of animal health, plant health, and food safety.

Its organizational structure includes a steering committee responsible for guiding the functioning of the committee and coordinating the implementation of the agreed actions. In accordance with Article 8 of the Regulation on the functioning of the CC-RAM (18), the chair and the secretariat are held by the competent authority of SENASAG, while the vice-chair is held by a representative of the productive sector, promoting a participatory governance scheme that articulates the public and private sectors.

The CC-RAM is composed of representatives from public institutions, producer organizations, professional associations, distributors of agricultural inputs, the pharmaceutical and pesticide industries, trade associations, academia, and other relevant stakeholders linked to agricultural production and the agri-food chain. This composition seeks to ensure broad representation of the sectors involved in AMR containment in the field of food and agriculture, while also promoting shared responsibility between competent authorities and productive sectors.

To address the specific challenges of each production system, the Committee establishes technical working groups by production sector, including poultry, swine, cattle, camelids, agriculture, and aquaculture. These groups are responsible for analyzing sector-specific issues, proposing technical measures, and developing work plans aligned with sectoral priorities and with the objectives of the NAP for AMR containment.

In addition, the CC-RAM’s regulatory framework enables the Committee to invite expert professionals, research centers, or specialized institutions—including representatives from other sectors, such as human health and the environment—to act as advisors to the CC-RAM and its technical working groups when required (18).

The functioning of the CC-RAM is based on regular meetings (every 3 months), complemented by extraordinary sessions, when necessary, technical coordination mechanisms, and consensus-based decision-making processes among its members. This scheme facilitates the exchange of information, strengthens articulation between public and private stakeholders, and promotes the implementation of the One Health approach in the containment of AMR within the agricultural sector.

### 3.5 Differentiating elements of the CC-RAM

The Committee introduces a formalized sectoral approach within the agricultural sector, representing a shift from exclusively multisectoral models, which in practice often face operational challenges when sectors do not have consolidated coordination mechanisms. Its main distinguishing feature is that it has its own regulatory backing, which provides institutional stability and clarity of mandate. In addition, it is intended to enable the agricultural sector to participate in future multisectoral settings as an organized bloc, with previously agreed positions, helping to avoid fragmented or uncoordinated interventions.

The CC-RAM is composed of representatives from public and private institutions involved in animal and plant-based food production, where all members of the Committee have the opportunity to provide input and make decisions regarding the actions to be implemented. This arrangement, together with the structure in which the chair of the Committee is held by the public sector and the vice-chair by the private sector, may reflect a significant degree of political and technical commitment to the decisions and actions taken, which may increase the likelihood of success of the actions implemented under the Committee. The model combines the leadership of the competent sanitary authority with the active participation of the productive sector, the pharmaceutical industry, academia, and professional associations, among others, and establishes technical working groups specialized by species or production sector. This facilitates addressing specific issues related to antimicrobial use and AMR with greater technical depth and based on the national context. In this sense, the CC-RAM does not replace multisectoral coordination, but rather it intends to strengthen it by first consolidating internal sectoral governance.

Committee meetings have been held regularly, demonstrating the support of the Ministry of Rural Development and Land (now the Ministry of Productive Development, Rural Affairs and Water) and the Executive Directorate of SENASAG as guarantor institutions, as well as the commitment of each participating entity. This also supports continuity of the work agenda and the delivery of results.

### 3.6 Results of CC-RAM implementation

Since its formalization in November 2023 and following its first ordinary meeting in April 2024, the CC-RAM held 11 sessions through December 2025, with between 26 and 45 participants each, consolidating a sectoral coordination space with regular functioning. This process has made it possible to progressively advance in the institutionalization of the AMR agenda within the food and agriculture sector in Bolivia.

The results presented below document the institutional process of establishing and operating the CC-RAM, and do not seek to evaluate the impact of its actions on the AMR situation in the country.

Among the main results achieved from the implementation process are:

- *Institutional formalization of the coordination mechanism*, through the approval of the Regulation on the functioning of the CC-RAM by means of SENASAG Administrative Resolution No. 383/2023.
- *Establishment of technical working groups by species and production sector*, enabling the specific addressing of AMR-related challenges in different production systems (19, 20). The poultry working group stands out as an example due to its ongoing activity in training events.
- *Development of sectoral work plans for 2025*, aligned with the priorities of the agricultural sector, which will serve as a basis for the update and completion of the NAP on AMR within the agricultural sector.
- *Establishment of a permanent space for technical discussion and coordination*, which has progressively enabled the integration of AMR into the agendas of public institutions and productive sectors, promoting a shared understanding of the importance of responsible antimicrobial use and its implications for agricultural production, food safety, and public health.
- *Increased sectoral ownership of the AMR agenda*, strengthening institutional and productive sector commitment to containment measures and contributing to the future implementation of the NAP, supported by a broader base of informed and aligned stakeholders.
- *Expanded visibility and understanding of the AMR problem within the agricultural sector*, with a multiplier effect across technical and productive networks at the territorial level, facilitating AMR awareness that may contribute to future changes in antimicrobial use practices.
- *Increased participation of sectoral stakeholders*, which has strengthened the inclusive nature of the mechanism and the sector’s representativeness in discussions on AMR-related policies. The most recent addition was the camelid sector.
- *Strengthened institutional support*, evidenced by the recognition and participation of the Executive Director General of SENASAG in the process, which has contributed to legitimizing the functioning of the committee and positioning the AMR agenda as a priority within the agricultural sector.

Definition of priority technical actions through the establishment of *ad hoc* groups, including:

- expansion of AMR awareness among stakeholders involved in food production;
- initiation of technical discussions on the appropriate use of antimicrobials in animal production, with a focus on the gradual elimination of the use of antimicrobials as growth promoters;
- progress toward the implementation of a system for the control of antimicrobial use in animal and plant production;
- development of an AMR surveillance plan in healthy animals (to later be implemented by SENASAG).

On the other hand, some actions resulting from the operationalization of the CC-RAM are as follows:

- development of a sectoral AMR communication strategy, including awareness-raising activities;
- implementation of a seminar series on antimicrobial use in animal feed, with particular emphasis on the reduction of their use as growth promoters (with four modules and the participation of a total of 750 animal production stakeholders);
- analysis of the national inventory of antimicrobials and its commercialization dynamics; and
- preparation of the terms of reference and the IT architecture for the veterinary and agronomic electronic prescription system.

Overall, these advances demonstrate that the establishment of a formal sectoral mechanism can translate into concrete actions, structured planning, and greater coherence in the implementation of the national AMR agenda within the food and agriculture sector.

### 3.7 Observed enabling factors for the establishment and functioning of the CC-RAM

The experience of the CC-RAM makes it possible to identify a set of factors that may have facilitated its implementation and effective functioning, and that may be relevant for the replicability of this experience in other countries.

- Evidence-based prior diagnosis

The establishment of the CC-RAM was preceded by a technical diagnostic process through the application of the FAO-PMP-AMR, FAO-ATLASS, and ACT tools, which enabled the identification of concrete gaps in governance, regulation, and sectoral coordination.

This analysis highlighted governance and coordination gaps that informed discussions on the possible establishment of a sector-specific coordination mechanism.

- Regulatory and institutional backing

The formalization of the committee through SENASAG Administrative Resolution No. 383/2023 provided a clear mandate, defining objectives, functions, and organizational structure. This regulatory backing provides a formal mandate and institutional framework that may support continuity and stability over time.

- Leadership of competent authority

The leadership exercised by SENASAG, including the support and participation of its leadership, appears to have played an important role in driving the functioning of the committee, facilitating coordination among institutions, and positioning the AMR agenda as a priority within the agricultural sector.

- Active participation and shared responsibility of the private sector

The inclusion of representatives from the productive sector, including professional associations, the pharmaceutical and pesticide industries, and academia within the committee structure has promoted an approach of shared responsibility in AMR containment. This participation may have improved the feasibility and acceptance of the measures adopted, and facilitated the mobilization of technical and operational resources that complement the capacities of the public sector.

- Technical organization by production sector

The creation of technical working groups by production sector was intended to facilitate recognition of sector-specific characteristics and the development of tailored work plans.

- Early generation of results

The rapid production of concrete results—such as the development of sectoral plans, the definition of priority technical actions, and progress in initiatives such as the veterinary and agronomic electronic prescription system, a national surveillance plan in healthy animals, and awareness-raising activities—may have contributed to sustaining stakeholder engagement.

- Expansion of awareness and sectoral ownership of the AMR agenda

The establishment of a formal and regular space for discussion may have contributed to broadening the understanding of the AMR problem within the agricultural sector. Through this process, public and private stakeholders appear to have progressively integrated the issue into their technical and operational agendas, promoting greater alignment around AMR containment. Likewise, the message may have extended to the technical and productive networks linked to committee participants, potentially contributing to strengthening awareness and commitment at the field level.

- Multisectoral visibility of progress in the agricultural sector

The presentation of CC-RAM results and progress to other sectors involved in the AMR agenda, such as health and environment, has made it possible to demonstrate the organizational capacity of the agricultural sector and may have contributed to generating incentives to strengthen multisectoral coordination in the country.

- Sectoral approach as a basis for multisectoral coordination

The establishment of a coordination mechanism within the agricultural sector itself enables the sector to first consolidate dialogue, organization, and alignment among its stakeholders. This process could strengthen the sector’s capacity to participate in a more articulated and effective manner in multisectoral coordination mechanisms at the national level.

## 4 Lessons learned and actionable recommendations

The experience of implementing the CC-RAM makes it possible to identify a set of institutional and governance factors that have supported its functioning and consolidation. These elements, derived from the implementation process and the coordinated work between the public sector and productive stakeholders, may provide useful guidance for other contexts seeking to strengthen sectoral AMR governance.

- *Formalize sectoral coordination mechanisms through a legal instrument*. Having a regulatory framework that establishes the mandate, objectives, and structure of the sectoral mechanism provides institutional legitimacy, clarity of functions, and continuity over time, regardless of administrative changes.
- *Define clear leadership within the competent authority*. The leadership of the mechanism should rest with the competent sanitary authority of the agricultural sector, ensuring coherence with public policies, ability to convene, and articulation with other state institutions and stakeholders.
- *Ensure public sector leadership with active participation of the productive sector*. AMR is a public health challenge that requires state leadership; however, the active participation of the productive sector in governance and decision-making enables the construction of shared commitments, incorporation of the technical, economic, and operational dimensions of production, and enhances the feasibility and sustainability of implemented measures.
- *Strengthen sectoral organization prior to multisectoral coordination*. Having a previously organized agricultural sector, with defined priorities and established coordination mechanisms, allows the sector to participate in multisectoral AMR governance bodies with an articulated and representative position. This strengthens its influence in decision-making and facilitates more effective progress in the implementation of joint actions.
- *Establish technical working groups by production sector*. Organization by production sector makes it possible to recognize the specific characteristics of each subsector, identify particular challenges, and develop concrete action plans adapted to different production realities.
- *Define sectoral priorities and progressively align them with the NAP*. Having clear sectoral priorities allows for progress in concrete actions even before the formal approval of the NAP and subsequently facilitates the alignment and integration of these initiatives within the national strategy.

Building on this experience, an additional recommendation going forward is to maintain a focused and feasible agenda. Keeping the Committee’s work plan concise and centered on specific, actionable priorities—rather than attempting to address the full breadth of demands posed by AMR containment—allows for steady and secure progress. This should be complemented by the establishment of formal monitoring and evaluation mechanisms within the CC-RAM, enabling systematic tracking of progress achieved and the identification of emerging sectoral needs, thereby giving continuity and focus to the work agenda over time.

## 5 Limitations

This study presents several limitations that should be considered when interpreting its findings.

As noted in the positionality statement (Section 2.2), all authors participated in the ACT project as FAO personnel or under FAO-supported technical arrangements, which may introduce a bias in the results presented.

The results presented reflect the CC-RAM’s process of institutional organization through December 2025—including the prioritization of the development of a surveillance plan, progress toward a veterinary and agronomic electronic prescription system, and the formulation of a sectoral AMR communication strategy, among others—but do not capture the effect of these actions on the AMR situation in the country. Assessing such effects would require the development of impact indicators, which fall outside the descriptive scope of this study.

This analysis is based on a single-country case study, developed within the scope of a project whose regional support in Latin America and the Caribbean was limited to two countries, with actions implemented in each country responding to its own national context and needs. This limits the extent to which the findings can be generalized to other national contexts (especially countries outside Latin America), particularly considering that all countries face different institutional, political, and resource challenges.

On the other hand, concrete indicators to measure progress in AMR governance and other key management actions were not established from the outset of the project’s implementation. In this regard, the development and application of monitoring indicators and/or the periodic reapplication of the FAO assessment tools (FAO-PMP-AMR, FAO-ATLASS, and the ACT tool)—or others—could have provided a more objective, if partial, basis for tracking progress over time, including the specific contributions of the CC-RAM through the concrete actions defined, developed, and implemented within this committee.

## 6 Discussion

The results of the case analyzed highlight the importance of coordination as a central element in AMR containment. Countries in early stages of NAP implementation are recommended to begin by establishing robust multisectoral governance to achieve coordination across sectors (9). Similarly, multisectoral engagement has been identified as one of the most consistent predictors of reductions in AMR prevalence (9).

However, countries face multiple difficulties in establishing One Health governance mechanisms that enable intersectoral coordination, including entrenched sectoral silos, unequal power dynamics, and reliance on external funding (14).

These elements describe the case of Bolivia, where, although the importance of establishing a multisectoral mechanism has been a continuous concern, various factors have hindered, as of the period covered by this study (through December 2025), the formal establishment of a multisectoral coordination mechanism and the implementation of the NAP. Considering, in addition, the recommendation that countries advance AMR management under a progressive approach based on their specific contexts (9), the experience of Bolivia reflects the search for alternatives in response to the difficulties that may arise in consolidating multisectoral coordination mechanisms, particularly in contexts where clear regulatory backing is lacking or where articulation among institutions is limited, which in turn hinders the consolidation and sustainability of these mechanisms.

Should this situation persist, there is a risk that the sectoral coordination achieved through the CC-RAM could remain disconnected from the human health, environment, and other relevant sectors, limiting its contribution to a One Health governance response.

Without disregarding that strengthening the agricultural coordination could deepen the silo-based work commonly observed among countries, the CC-RAM was conceived, from the outset, as a sectoral mechanism for the agricultural sector intended to subsequently feed into a future multisectoral coordination mechanism once established. This is consistent with international guidance, which states that sector-specific activities and planning should be carried out within the framework of each sector, while recognizing the need for cross-sectoral collaboration, and promotes the establishment of sector-specific working groups or subcommittees connected to other sectors (21). In practice, the Committee’s functioning, together with its ability to invite external participants, when necessary, has already allowed it to share sectoral progress and experience with other sectors such as human health and the environment, reinforcing the One Health spirit embedded in the Committee, in line with the guidelines established by SENASAG through REGENSA. At the same time, this does not mean that the CC-RAM constitutes a One Health governance mechanism, since it does not currently include formal representation from all sectors involved (Figure 1).

**Figure 1:**
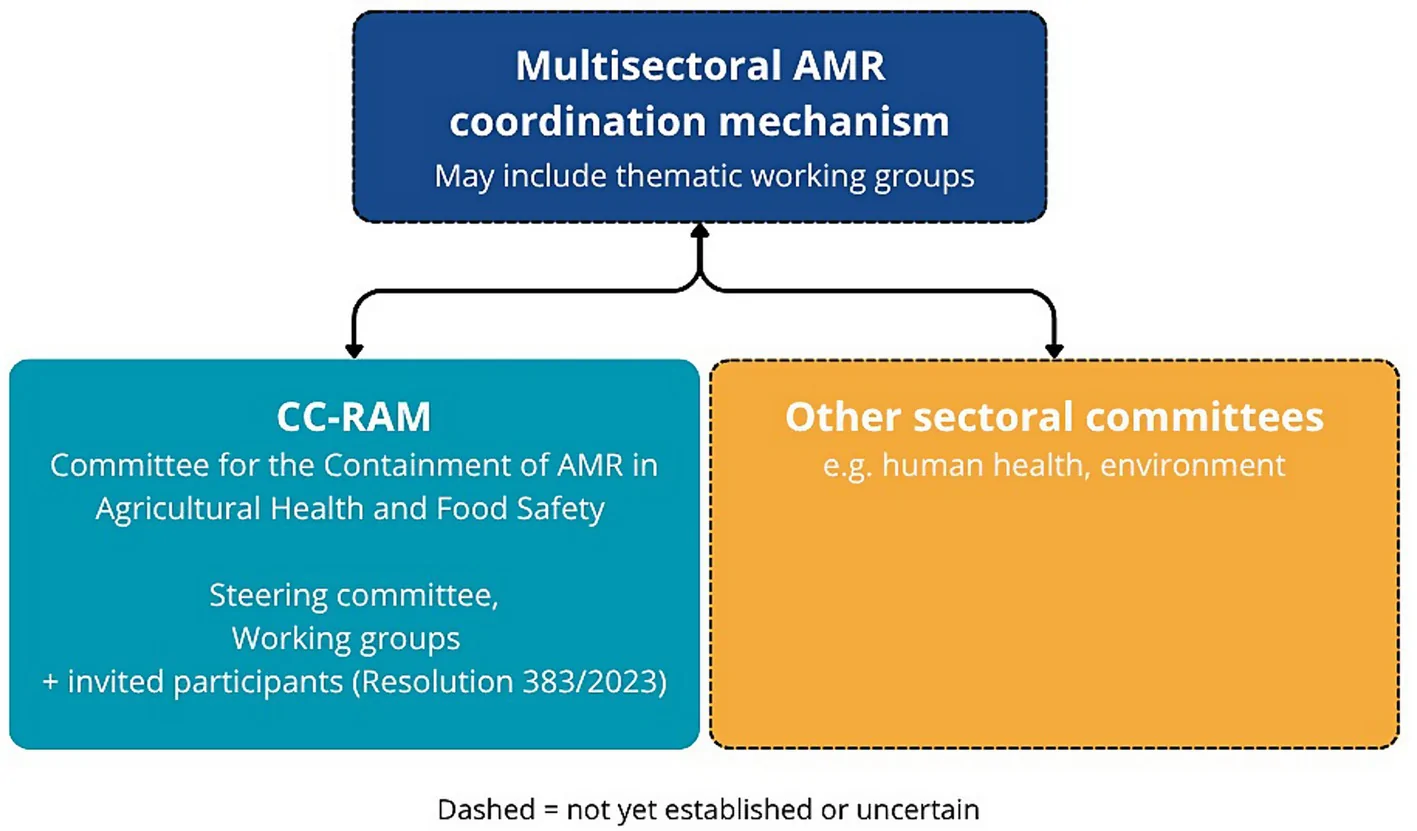
Proposed conceptual positioning of the CC-RAM within a One Health governance framework, whose coordination mechanism remains undefined.

The experience accumulated through the CC-RAM illustrates concrete achievements at the sectoral level. Within the agricultural sector, the CC-RAM has enabled the creation of a space for articulation that facilitates exchange among stakeholders, the definition of priorities, and the development of concrete actions; its success does not depend solely on the existence of formal structures, but also on political and institutional commitment, the technical engagement of stakeholders, and the effective functioning of the mechanism in practice. Likewise, stakeholders’ understanding of the critical importance of AMR has contributed to strengthening ownership of the issue and sustaining the continuity of the work undertaken. This has made it possible to advance in the implementation of measures and to contribute to their sustainability over time. These elements are linked to the challenges inherent to the One Health approach, in which AMR containment requires both coordination across different sectors and the development of specific capacities within each of them. In this regard, the experience of the CC-RAM highlights the importance of sectoral work and how it can contribute to organizing and strengthening the participation of the agricultural sector, as well as advancing the national AMR agenda within its sphere of action. This is reflected in the institutionalization of sectoral coordination mechanisms that articulate the public sector with productive stakeholders in the agricultural sector. These mechanisms not only improve technical dialogue and informed decision-making, but also promote trust and shared responsibility in the implementation of measures aimed at AMR containment.

A useful framework for situating this experience is provided by Clifford Astbury et al. (14), who identify six core dimensions of One Health governance—participation, leadership, decision-making, coordination, resourcing, and accountability—that should be considered when designing governance models, alongside guiding principles of transparency, sustainability, inclusivity, and equity. Applied to the CC-RAM, this framework suggests that the Committee has functionally operationalized several of these dimensions at the sectoral level: broad-based participation of public and private stakeholders, shared leadership between the competent authority and the productive sector, structured and consensus-based decision-making, and formal coordination anchored in a legal instrument. At the same time, this experience remains uneven across dimensions: resourcing continues to depend significantly on external project support, and the CC-RAM has yet to develop dedicated accountability structures, including monitoring and evaluation mechanisms to systematically track its progress and inform its continued adaptation. This is consistent with broader evidence suggesting that countries’ efforts have concentrated primarily on AMR policy design and implementation, while systematic monitoring and evaluation of policy effectiveness and cost-efficiency have remained comparatively weak, limiting opportunities to improve strategic design and implementation over time (22). Despite these gaps in resourcing and accountability, the CC-RAM’s accumulated experience across the remaining governance dimensions may serve as an input or foundation for the design and implementation of a future multisectoral coordination mechanism in Bolivia, without seeking to replace or substitute it.

An additional consideration relates to the sustainability of the CC-RAM beyond this initial stage of establishment. Evidence from global governance analyses suggests that countries often succeed in designing progressive policies and identifying the mechanisms required for their implementation, but frequently lack concrete follow-through and fail to demonstrate sustained action (22). The Committee’s establishment and initial functioning relied to a certain extent on the support provided by the ACT project. Low- and middle-income countries may be unable to secure sufficient funding on their own (22). In this context, finding funding for the continued operation of the CC-RAM, as well as maintaining an active, continuous agenda with clear objectives, may represent challenges now that the project has concluded (July 2026). Countries face multiple priorities, and although AMR currently remains part of the agricultural sector’s agenda, there is a risk that this priority could lose ground relative to other national concerns, as such agendas remain subject to countries’ social, economic, and political circumstances. In this sense, establishing the CC-RAM may have represented the more straightforward part of this process, while ensuring its sustained and effective functioning over time constitutes the greater challenge. However, some elements suggest a degree of resilience in this regard.

Sustained participation over time provides a first signal of resilience: meetings have been maintained with regularity, and while the first meeting counted 26 participants, subsequent meetings have consistently gathered no fewer than 30, reaching up to 45, along with an increase in the number of participating institutions. On the other hand, the private sector has contributed to the financing of Committee meetings, which points to an emerging avenue for sustained financing. The Committee’s meetings can also be, and have on occasion been, held virtually, further reducing the operational costs associated with its continued functioning. Most importantly, the CC-RAM’s legal backing through SENASAG’s Administrative Resolution provides institutional continuity independent of external funding, mandating the Committee’s continued functioning and establishing an obligation for SENASAG to dedicate working hours to it.

Taking into account the advantages and risks discussed above, the case analyzed contributes to the discussion on AMR governance approaches, highlighting the role of sectoral strengthening as a key element for advancing effectively in the implementation of national AMR containment agendas in the agricultural sector and its possible contribution to the functioning of the multisectoral governance under the One Health approach. In this regard, the Bolivian experience highlights the advantages of having previously organized agricultural sector, with internal structures for coordination, representation, and priority-setting, as a supporting element for the development of multisectoral articulation mechanisms.

It is also important to note that the results presented in this article refer primarily to process-related achievements of the CC-RAM, rather than to the impact of these achievements on the AMR situation in the country. This does not diminish the relevance of governance as a foundational element for advancing AMR containment; rather, it points to the need for a longer observation period and dedicated impact indicators to establish with greater certainty the extent to which Bolivia’s governance efforts, including those of the CC-RAM, have translated into measurable progress on AMR across the different sectors involved.

To the best of the authors’ knowledge and based on a review of the available peer-reviewed literature, this represents a novel experience in Latin America. No other initiative with similar characteristics was identified that formally institutionalizes, through a regulatory instrument, a sector-specific coordination mechanism for AMR governance that brings together public and private stakeholders in setting the AMR agenda and supporting the implementation of coordinated actions within the agricultural sector.

Comparable sector-specific arrangements can be found elsewhere in Latin America, although their institutional design differs from that of the CC-RAM. In Brazil, the *Comissão sobre Prevenção da Resistência aos Antimicrobianos* (CPRA) operates within the Ministry of Agriculture and Livestock as a consultative body responsible for planning, monitoring and evaluating the implementation of the agricultural component of the national AMR action plan (PAN-BR AGRO), as well as proposing and monitoring measures relating to AMR prevention and control, surveillance and the prudent use of antimicrobials (23). Its formal membership consists of representatives of different units of the Ministry, while representatives of other public institutions and private entities may be invited to participate in specific meetings on an *ad hoc* basis. Mexico provides a different institutional model. The national *Comité Técnico sobre Resistencia a los Antimicrobianos* brings together, among others, health, agriculture and environmental authorities and is supported by three specialized subcommittees addressing human, animal and environmental AMR, respectively. The *Subcomité sobre Resistencia a los Antimicrobianos Animal* (SCRAM-V) is thus institutionally embedded within the national multisectoral governance mechanism and contributes to monitoring implementation of the respective national AMR strategy, including through the development of indicators and activity reports. The SCRAM-V may also invite representatives from other public or private institutions to participate in its meetings, although such invited participants have voice but no vote (24). These examples illustrate different configurations through which sector-specific coordination may contribute to broader AMR governance: a dedicated agricultural mechanism linked to the national action plan, as in Brazil, and a specialized sectoral body formally embedded within an overarching multisectoral structure, as in Mexico. The Bolivian experience represents a further configuration, in which a sector-specific mechanism with both public and private stakeholders represented in its steering body has been developed and institutionalized in advance of the establishment of a fully defined national multisectoral AMR coordination mechanism.

Building on this experience, the CC-RAM may provide useful insights for other countries facing similar challenges seeking to strengthen their institutional mechanisms for AMR containment in the agricultural sector, and could also serve as a reference for strengthening and advancing in the AMR coordination of other sectors, as well as for advancing multisectoral coordination more broadly.

## Footnotes

1 FAO Progressive Management Pathway for Antimicrobial Resistance (FAO-PMP-AMR) | Antimicrobial Resistance | Food and Agriculture Organization of the United Nations.

2 ATLASS | Antimicrobial Resistance | Food and Agriculture Organization of the United Nations.

3 The ACT Tool assesses the level of incorporation of relevant Codex Alimentarius standards on AMR into national legislation. It has been incorporated into the 2026 version of the FAO methodology for the analysis of AMR legislation in the food and agriculture sectors (forthcoming).
